# Can Leptin/Ghrelin Ratio and Retinol-Binding Protein 4 Predict Improved Insulin Resistance in Patients with Obesity Undergoing Sleeve Gastrectomy?

**DOI:** 10.1007/s11695-022-06296-2

**Published:** 2022-10-06

**Authors:** Mohamed Hany, Hala M. Demerdash, Ann Samy Shafiq Agayby, Mohamed Ibrahim, Bart Torensma

**Affiliations:** 1 Department of Surgery, Medical Research Institute, Alexandria University, 165 Horreya Avenue, Hadara, 21561 Alexandria Egypt; 2 Clinical Pathology, Alexandria University, Alexandria, Egypt; 3 Medical Research Institute, Alexandria University, Alexandria, Egypt; 4 Leiden University Medical Center (LUMC), Leiden, The Netherlands

**Keywords:** HOMA-IR, Metabolic biomarkers, Prediction model, ROC curve, Sleeve gastrectomy

## Abstract

**Introduction:**

Obesity is associated with metabolic syndrome (MBS), a cluster of components including central obesity, insulin resistance (IR), dyslipidemia, and hypertension. IR is the major risk factor in the development and progression of type 2 diabetes mellitus in obesity and MBS. Predicting preoperatively whether a patient with obesity would have improved or non-improved IR after bariatric surgery would improve treatment decisions.

**Methods:**

A prospective cohort study was conducted between August 2019 and September 2021. We identified pre- and postoperative metabolic biomarkers in patients who underwent laparoscopic sleeve gastrectomy. Patients were divided into two groups: group A (IR < 2.5), with improved IR, and group B (IR ≥ 2.5), with non-improved IR. A prediction model and receiver operating characteristics (ROC) were used to determine the effect of metabolic biomarkers on IR.

**Results:**

Seventy patients with obesity and MBS were enrolled. At 12-month postoperative a significant improvement in lipid profile, fasting blood glucose, and hormonal biomarkers and a significant reduction in the BMI in all patients (p = 0.008) were visible. HOMA-IR significantly decreased in 57.14% of the patients postoperatively. Significant effects on the change in HOMA-IR ≥ 2.5 were the variables; preoperative BMI, leptin, ghrelin, leptin/ghrelin ratio (LGr), insulin, and triglyceride with an OR of 1.6,1.82, 1.33, 1.69, 1.77, and 1.82, respectively (*p* = 0.009 towards *p* = 0.041). Leptin had the best predictive cutoff value on ROC (86% sensitivity and 92% specificity), whereas ghrelin had the lowest (70% sensitivity and 73% specificity).

**Conclusion:**

Preoperative BMI, leptin, ghrelin, LGr, and increased triglycerides have a predictive value on higher postoperative, non-improved patients with HOMA-IR (≥ 2.5). Therefore, assessing metabolic biomarkers can help decide on treatment/extra therapy and outcome before surgery.

**Graphical Abstract:**

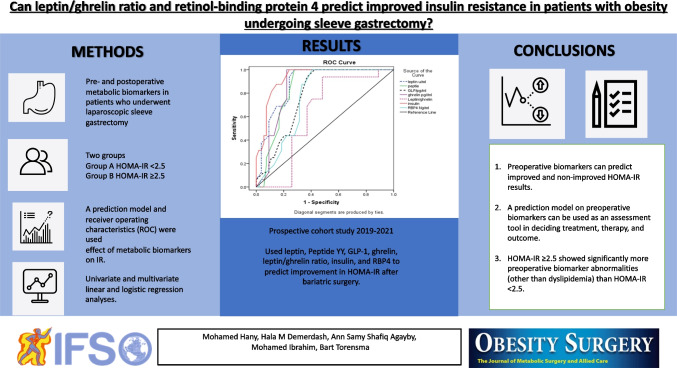

## Introduction

Obesity is associated with metabolic syndrome (MBS), which is a cluster of components including central obesity, insulin resistance (IR), dyslipidemia, and hypertension [1]. MBS increases the risk of type 2 diabetes mellitus (T2DM) by approximately fivefolds [2].

IR is the major risk factor in the development and progression of T2DM in obesity and MBS [3, 4]. IR is defined as a state where insulin is required more than the normal amounts to obtain a quantitatively normal response [2, 5].

The hormonal regulation of food intake and energy hemostasis is influenced by the antagonistic action of ghrelin and leptin, respectively [6]. Ghrelin, a stomach-derived hormone, promotes food intake and is involved in lipogenesis and insulin sensitivity [7]. It inhibits insulin-producing β-cells and somatostatin-producing D-cells in the pancreas [8]. Leptin is secreted mainly by adipose tissue in amounts proportional to fat stores and a lesser extent, by the stomach. It is a pro-inflammatory adipokine that stimulates satiety and activates catabolic pathways and improves the insulin sensitivity of the peripheral tissues [6, 9].

The interaction between both hormones is reciprocally regulated during the physiological response to food intake [10]. Therefore, it is logical to combine these two hormones, and the ratio of their concentrations (leptin/ghrelin ratio) is described as a hunger signal [11].

Then, the retinol-binding protein 4 (RBP4), which is an adipokine released mainly by the adipose tissue and liver [12]. It facilitates the transport of retinol and fatty acids in circulation. Previous studies have proposed the involvement of RBP4 in IR through its pro-inflammatory functions associated with increased expression of monocyte chemoattractant protein-1 in adipocytes and suppressed peripheral expression of glucose transporter 4 (GLUT-4) in skeletal muscles [12, 13].

Furthermore, several hormones are involved in the regulation of glucose homeostasis, including the incretin of glucagon-like peptide-1 (GLP-1) and peptide YY (PYY).

GLP-1 is expressed in the pancreatic α-cells and L cells of the intestinal mucosa [14], is secreted in response to food ingestion, and plays a role in slowing gastric emptying and soothing postprandial glycemia [15, 16]. PYY affects insulin sensitivity and is co-secreted with GLP-1 and decreases gastrointestinal motility, and inhibits pancreatic exocrine secretion through suppression of cholecystokinin and secretin [17, 18].

Looking more deeply into baseline metabolic traits on the response to LSG, studies showed that obesity-associated energy metabolic disruption was illustrated by the downregulation of the Krebs cycle and reduced oxidation of ketone bodies [19]. However, in the short-term period after surgery, most intermediate and end products of β-oxidation and ketogenesis upsurge were seen after LSG, whereby fatty acid levels, triglycerides, total cholesterol, and LDL cholesterol are frequently raised in patients with obesity [20].

Lemaitre et al. proposed that a preoperative lipid profile with higher triglycerides and phospholipids with long-chain fatty acids could assess the probability of T2DM remission after surgery [21]. Also, circulating amino acids (AAs) were the most characteristically changed metabolite, differentiating patients that had T2DM remission from those with persistent insulin resistance after LSG [22, 23]. Kwon et al. depicted not only an early decline in AAs improved insulin resistance but also both branched-chain AA and aromatic amino acids had a role in improved insulin resistance 3 months after LSG [22].

So, this study aimed to develop a prediction model using metabolic biomarkers and their effect on HOMA-IR, so if we can predict preoperatively whether a patient with obesity would have an improved or non-improved IR after bariatric surgery and if this would help improve treatment decisions.

## Methods

### Study Design

This was a prospective cohort study conducted between August 2019 and September 2021 at the Medical Research Institute, Alexandria University, Egypt. Written and oral informed consents were obtained from all the patients, and data were analyzed anonymously. The study was conducted in accordance with the principles of the Declaration of Helsinki and approved by the ethical committee board.

### Patient Selection

Patients aged between 18 and 65 years with American Society of Anesthesiology (ASA) classes 1–3 were screened according to the National Institutes of Health (NIH) criteria [24] before laparoscopic sleeve gastrectomy (LSG) surgery. Components of MBS were used according to the International Diabetes Federation (IDF) modified criteria (Appendix 1).

### Exclusion Criteria

Patients with chronic inflammatory bowel disease, malignancy, psychiatric illnesses, secondary causes of obesity such as Cushing syndrome, patients taking antipsychotic medication, females receiving contraceptive pills, pregnant or lactating mothers, gastrointestinal diseases such as previous surgery for gastric or duodenal ulcer, and systemic diseases such as a malignant tumor or autoimmune disease were excluded.

### Laparoscopic Sleeve Gastrectomy (LSG)

LSG was performed by the same team throughout the study. Dissection was started 6 cm from the pylorus (antrum preserving) up to the gastroesophageal junction, followed by gastric transaction over a 40F bougie through sequential stapler firings.

### HOMA-IR Selection

Two groups were created: HOMA-IR < 2.5 (group A, improved IR) and HOMA-IR ≥ 2.5 (group B, not improved IR), considered to have IR [25]. Postoperative, we tested the changes in levels of HOMA-IR again to identify the number of patients with improved insulin sensitivity.

### Leptin/Ghrelin Ratio (LGr)

LGr was calculated as leptin in nanogram per milliliter multiplied by 10 × 3 and divided by ghrelin in picograms per milliliter.

### Laboratory Investigations

Peripheral blood samples were collected before and 12 months postoperatively to measure the fasting levels of the hormones (ghrelin, leptin, RBP4, and insulin) and postprandial levels of GLP-1 and PYY. Serum samples were allowed to clot at 18–22 °C degrees for 30 min and then centrifuged at 4000 rpm for 10 min at 4 °C.

### Hormonal Measurements

All measurements were analyzed according to standardized operating procedures. Lipid profile was determined using Hitachi 7180 Biochemistry Automatic Analyzer (Hitachi, Japan), and hormones were determined using ELISA (EIA-2935) (DRG International, Inc. Springfield NJ, USA) (Appendix 2).

### Blood Sampling Sequence

Blood sampling was performed 1 week prior to surgery. First, fasting samples were obtained to measure the levels of metabolic biomarkers, including leptin, ghrelin, RBP4, insulin, total cholesterol, HDLs, LDLs, FBS, HOMA-IR, and triglycerides. The patients were then provided with a standard meal (300 kcal) containing pasta (30 g), ground lean meat (30 g), olive oil (5 g), almonds (*n* = 6), yogurt (80 g), and dried prune (*n* = 1), providing 45% carbohydrates, 20% protein, and 35% fat. The meal duration was 15 min. Blood samples were collected for the postprandial determination of GLP-1 and PYY after ingestion and 120 min after the standard meal.

### Statistical Methods

Descriptive and inferential statistics were used for analyses. All data were tested for normality using the Kolmogorov–Smirnov test, Q-Q plot, and Levene’s test. Categorical variables are expressed as numbers and percentages. Continuous normally and non-normally distributed variables are presented as means and standard deviations (SDs) and medians and interquartile ranges (for skewed distributions), respectively. When appropriate, categorical variables were tested using Pearson’s chi-square test or Fisher’s exact test. Normally distributed continuous data were tested with dependent samples using the Student’s *t*-test for pre- and postoperative results. For skewed (non-parametric) data, the Wilcoxon signed-rank test was used.

Analysis of the receiver operating characteristics (ROC) curve was done to determine the optimal threshold value and predictive ability, as well as the area under the curve (AUC).

Predictors were evaluated using univariate and multivariate linear and logistic regression analyses. All independent variables, counting more than ten events with *P*-values < 0.1 were eligible for multivariable analysis, which was achieved through backward selection. The optimal prediction model was evaluated with a 2 log-likelihood. *P*-values < 0.05 were considered statistically significant. Statistical analyses were performed using R (version 4.0.4) packages.

### Sample Size

G*power version 3.1.9.5 was used for sample size calculation. Our main end-point was the pre- and postoperative change in HOMA-IR of a patient. Reporting a minimum of 50% changes, a conservative mean difference of 0.8 to 1 point on HOMA-IR and SD of 2.0 would be needed. With an alpha of 0.05 and a beta > 0.8, we needed 52 patients. Considering a 10% possible loss to follow-up, a minimum of 57 patients should be recruited.

## Results

### Baseline Characteristics

A total of 70 patients with obesity and MBS who underwent an LSG were enrolled in this study, with 25 (35.72%) men and 45 (64.28%) women, and a mean age ± SD of 33.8 ± 11.45 years. The preoperative and 12 months postoperative BMIs (mean ± SD) were 44.9 ± 6.8 and 31.9 ± 4.7 kg/m^2^, respectively. The 12-month postoperative % excess weight loss ((%EWL) (mean ± SD)) was 59.9 ± 15.7 (Table 1).

**Table 1:** Laboratory investigations of all studied patients preoperative and 12 months postoperative

	Preoperative	Postoperative	*P* value
BMI (kg/m^2^)	45.17 ± 6.61	31.98 ± 4.52	0.001*
HOMA-IR	5.96 ± 3.61	2.82 ± 2.86	0.003*
Leptin (ng/mL)	53.33 ± 13.93	23.07 ± 12.65	0.001*
Peptide YY (pg/mL)	188.87 ± 54.03	234.06 ± 70.58	0.006*
GLP-1 (pmol/L)	3.57 ± 1.05	4.45 ± 0.81	0.001*
Ghrelin (pg/mL)	328.21 ± 102.22	232.53 ± 22.97	0.002*
Leptin/ghrelin ratio	170.24 ± 47.39	99.92 ± 56.76	0.001*
Insulin (mIU/L)	23.98 ± 14.45	14.88 ± 13.46	0.001*
RBP4 (ng/mL)	43.36 ± 15.16	34.43 ± 17.73	0.001*
T. cholesterol (mg/dL)	268.96 ± 54.57	159.81 ± 30.77	0.001*
Triglycerides (mg/dL)	175.93 ± 77.96	90.36 ± 41.88	0.002*
HDL cholesterol (mg/dL)	49.89 ± 7.48	40.69 ± 7.84	0.003*
LDL cholesterol (mg/dL)	182 ± 49.63	100.63 ± 25.02	0.005*
FBS (mg/dL)	103.03 ± 9.47	89.51 ± 16.23	0.004*

Results are expressed as mean ± SD, *P* value significant ≤ .05

*FBS*, fasting blood sugar

Common obesity-associated medical conditions identified in the patients were asthma (5.5%), diabetes (11%), hypertension (7.4%), hypothyroidism (16.6%), and history of cancer (3.7%).

### Levels of Metabolic Biomarkers

#### Postprandial Values

Significant changes were observed in GLP-1 and PYY levels pre- and 12 months after surgery (*p* = 0.006) (Table 1).

#### Fasting Values

Significant (*p* < 0.05) changes were observed between the pre- and 12-month postoperative levels of the metabolic biomarkers for leptin, ghrelin, insulin, total cholesterol, HDLs, LDLs, FBS, HOMA-IR, and triglyceride levels, respectively (Table 1).

### Levels of Leptin/Ghrelin Ratio (LGr) and RBP4

Significant (*p* = 0.001) changes were observed between the pre-and 12-month postoperative levels of LGr and RBP4 (Table 1).

### HOMA-IR Groups

Preoperatively, group A (IR < 2.5) contained 14 patients (20%), and group B (IR ≥ 2.5) had 56 patients (80%). Postoperatively (12 months), 54 (77.1%) patients were in group A and 16 (22.9%) in group B (*p* = 0.001) (Table 2).

**Table 2:** Classification of patients according to HOMA-IR

	HOMA-IR (preoperative)		HOMA-IR (postoperative)	
	No	%	No	%
Group A ** <** 2.5 IR	14	20.0	54	77.1
Group B ** ≥ **2.5 IR	56	80.0	16	22.9
Total	70	100.0	70	100.0
*P* value	0.001*			

Group B had a significantly higher preoperative BMI and excess weight than group A (*p* = 0.008, *p* = 0.002).

In addition, group B (HOMA-IR ≥ 2.5) had significantly more preoperative biomarker abnormalities (other than dyslipidemia) than group A, including leptin, ghrelin, PYY, insulin, RBP4, and LGr (Table 3).

**Table 3:** Comparison between preoperative clinical and laboratory parameters in groups A and B according to their postoperative HOMA-IR

	Postoperative HOMA-IR		*P* value
	Group A < 2.5 IR *n* = 54	Group B ≥ 2.5 IR *n* = 16	
Age	34.91 ± 11.57	32.69 ± 11.32	0.501
Sex (M/F)	(21/33)	(4/12)	0.238
BMI (kg/m^2^)	44.04 ± 6.97	48.97 ± 3.03	0.008*
Excess weight** (**kg)	57.70 ± 20.76	75.25 ± 10.30	0.002*
leptin (ng/mL)	49.13 ± 12.13	67.49 ± 9.76	0.001*
Peptide YY (pg/mL)	180.22 ± 58.56	169.97 ± 49.20	0.003*
GLP-1 (pmol/L)	3.53 ± 0.63	3.09 ± 0.30	0.032*
Ghrelin (pg/mL)	325.4 ± 111.3	337.62 ± 64.31	0.021*
Insulin (mIU/L)	18.73 ± 11.25	41.69 ± 8.93	0.001*
RBP4 (ng/mL)	40.63 ± 15.31	52.56 ± 10.59	0.005*
Leptin/ghrelin ratio	168.30 ± 48.4	176.43 ± 44.5	0.026*
T. cholesterol (mg/dL)	257.41 ± 54.78	307.94 ± 31.52	0.001*
Triglycerides (mg/dL)	160.65 ± 75.18	227.50 ± 65.66	0.002*
HDL cholesterol (mg/dL)	51.43 ± 7.50	44.69 ± 4.63	0.001*
LDL cholesterol (mg/dL)	171.11 ± 50.00	218.75 ± 25.16	0.001*
FBS (mg/dL)	101.15 ± 9.38	109.38 ± 6.79	0.002*

Results are expressed as mean ± SD; *P*-values ≤ .05 are significant

### Multiple Logistic Regression Analysis of Preoperative Risk Factors Predicting Change in Postoperative HOMA-IR ≥ 2.5

A univariate regression analysis was performed on every preoperative variable. After backward selection, the best-fitted prediction model with a multiple logistic regression was developed using BMI, leptin, ghrelin, LGr, insulin, and triglyceride which showed significant effects on the change in HOMA-IR ≥ 2.5 (*p* = 0.009 towards *p* = 0.041).

Therefore, the higher the preoperative levels, the greater the change in HOMA-IR ≥ 2.5 is reached. RBP4 had no significant effect in the multiple logistic regression but was significant in the univariate regression analysis (*p* = 0.004) (Table 4).

**Table 4:** Multiple logistic regression analysis of preoperative risk factors predicting change in postoperative HOMA-IR

Dependent variable: postoperative HOMA-IR ≥ 2.5	Odd’s ratio	*P* value
BMI	1.6	.017*
Leptin	1.82	.009*
Ghrelin	1.33	.041*
Leptin/ghrelin	1.69	.021*
Insulin	1.77	.017*
Triglycerides	1.82	.026*

### Receiver Operating Characteristics (ROC)

ROC curve analysis with AUC estimations was performed to determine the optimal cutoff value and predictive ability of selected metabolic biomarkers for the best value on preoperative assessments.

**Fig. 1:**
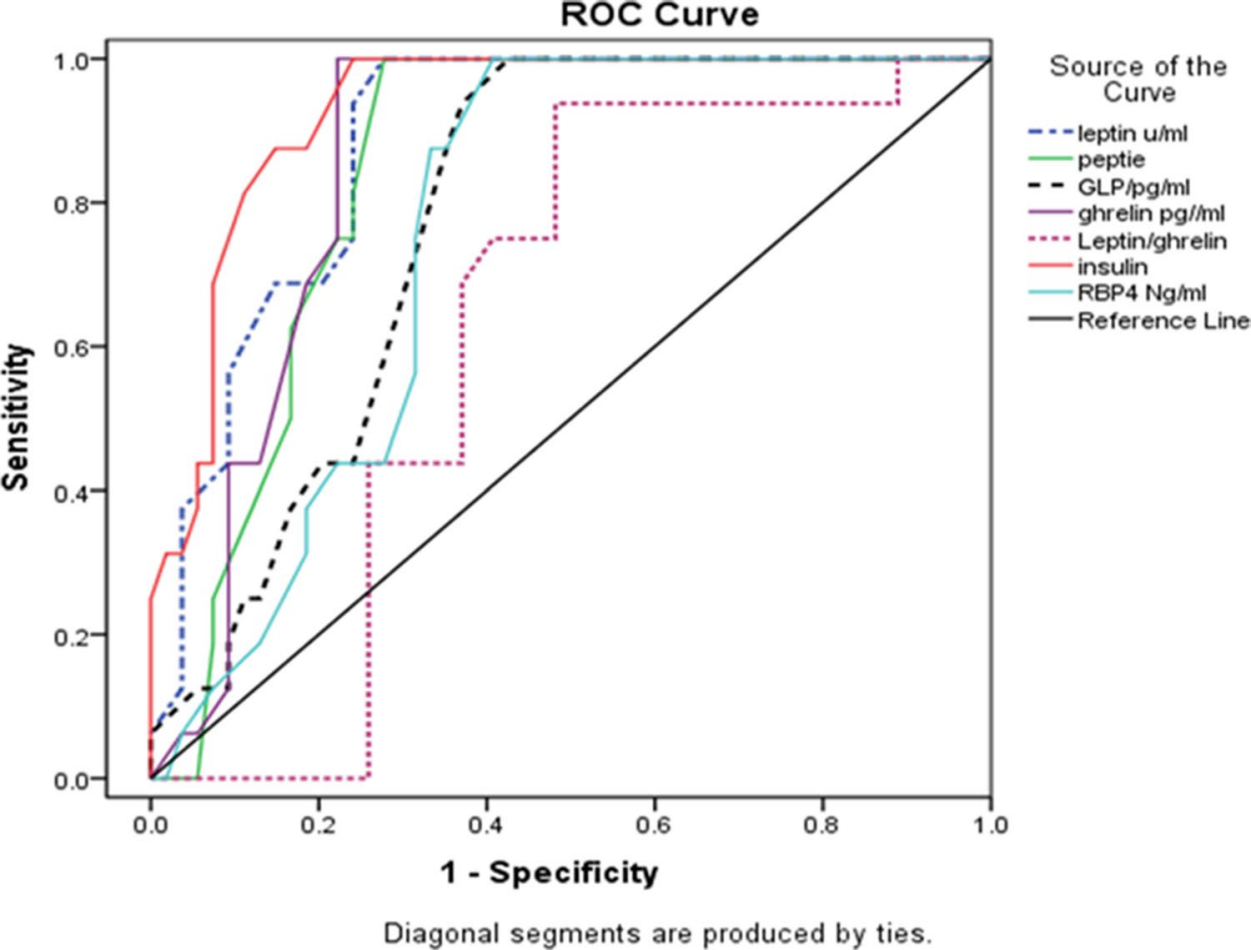
Receiver operating characteristics (ROC) curve analysis. Using leptin, peptide YY, GLP-1, ghrelin, leptin/ghrelin ratio, insulin, and RBP4 to predict improvement in HOMA-IR after bariatric surgery

- The optimal LGr AUC was 0.742 with 79% sensitivity (sens) and 74% specificity (spec).
- Ghrelin AUC was 0.710 with 73% sens and 70% spec.
- Leptin AUC was 0.88 with a sens of 92.0% and spec of 86%.
- RBP4 AUC was 0.76 with 75% sens and 70% spec.
- The best cutoff values of all variables, GLP-1, insulin, and peptide YY are presented in Table 5 (Fig. 1).

**Table 5:** Area under the curve and cutoff values, sensitivity, specificity, and accuracy of selected biomarkers to predict improvement in HOMA-IR after bariatric surgery

Variable	Area	Cutoff value	*P* value	Sensitivity	Specificity	Accuracy
Leptin	0.884	55	0.0004	92.0	86.0	89.0
Peptide YY	0.812	170	0.0131	80.0	77.0	78.0
GLP-1	0.771	3.3	0.011	73.0	71.0	72.0
Ghrelin	0.710	325	0.014	73.0	70.0	71.0
Leptin/ghrelin	0.742	169	0.028	79.0	74.0	75.0
Insulin	0.928	25	0.0001	95.0	88.0	92.0
RBP4	0.760	45	0.002	75.0	70.0	72.0

## Discussion

This study analyzed 70 patients with obesity who presented with MBS and an indication for bariatric surgery. An evaluation of HOMA-IR levels with a cutoff value of 2.5 was performed, and a predictive model was developed using preoperative biomarkers based on the outcome of the HOMA-IR response.

Twelve months after surgery, there was a significant reduction in BMI in all patients (*p* = 0.008), and HOMA-IR significantly decreased in 57.14% of patients.

Consequently, postoperative group A (HOMA-IR < 2.5) represented improved patients, which increased to 77.1%, whereas group B (HOMA-IR ≥ 2.5) represented 22.9%. This improvement was attributed to enhanced insulin sensitivity after surgery. Furthermore, there was a significant postoperative improvement in lipid profile, fasting blood glucose, and hormonal biomarkers. The results obtained in our cohort were in line with previous studies on bariatric surgery patients [6, 11]. Our study had a %EWL of 59.9 ± 15.7. When we look in the literature at studies with also 1-year outcomes, we see results with a wide range from lower and higher %EWL of 43.8 ± 18.9 to 43.6% EWL [26, 27] towards 74.3 ± 2.6 at 12 months [28]. Possible explanations for our results are that this cohort had only 70 patients, which may not have sufficient dispersion in overall responders on weight loss and the associated %EWL and, therefore, a somewhat lower outcome.

Also, possible slower responders after LSG at 1 year, patients still in the weight loss phase and did not reach the target %EWL is a possible explanation.

Nevertheless, most of this cohort reached the Reinhold criteria ≥ 50%EWL as a successful weight loss [29].

### Leptin/Ghrelin Ratio (LGr)

LGr was significantly higher in group B (*p* = 0.026), implying that an elevated ratio was associated with the non-improved patients, and this could be used to identify patients with resilient IR after bariatric metabolic surgery. Few studies have investigated LGr relationship with obesity-associated IR, particularly after weight loss [10, 30]. A study by Sitar-taut et al. [30] found that LGr was significantly higher in overweight and diabetic patients or patients with metabolic syndrome and obesity. They reported that LGr correlated with BMI (*r* 0.304, *p* = 0.018), and diabetes (*r* 0.318, *p* = 0.013). Furthermore, LGr showed a good predictive ability in the improvement of HOMA-IR after bariatric surgery in this study and can be added to prediction model tools.

Future studies with bigger cohorts, longer follow-ups, and other bariatric procedures are warranted to verify the power of the ratio versus the values of leptin and ghrelin alone. Nevertheless, our results may be of added value in preoperative screening and assessment and can help in deciding best suitable procedures for patients.

### Predictive Modeling for the Future

In every medical setting, the question remains, what would be the best procedure for my patients? Bariatric metabolic surgery is effective for patients with obesity [16, 31]. A multiple logistic regression analysis was performed to determine the preoperative predictors, which resulted in that preoperatively biomarkers were significantly increased on the values of BMI, leptin, ghrelin, LGr, and increased triglycerides who all have a predictive value for higher postoperative HOMA-IR ≥ 2.5. This positive finding revealed that patients with lower weight loss after surgery (retained higher BMI) and consequently less reduction in metabolic biomarkers (retained higher biomarkers) have an increased chance of non-improved IR and therefore are less healthy after LSG. In addition, previous studies have reported the significance of LGr as a combination of two hormones on the body’s metabolic status, suppressing hunger sensation [10, 30, 32]. Furthermore, this study supports the theory that both leptin and ghrelin play substantial roles in the development of hyperinsulinemia and IR in individuals with obesity. Since the values are taken preoperatively, this minimally invasive blood test can help physicians better and prepare patients for specific treatments, for example, extra training and assessments (e.g., dialectical behavior therapy) [33] or changing treatment procedures (such as a two-step procedure with SADI_S instead of a one-step procedure or placement of a band). Bariatric surgery should be more personalized in the treatment of patients with obesity. This type of predictor is increasingly one of the key points in achieving this.

### ROC

Regarding the best cutoff value on the ROC for the prediction of preoperative values, this study evaluated several metabolic biomarker levels. All were close to the preoperative HOMA-IR < 2.5 value. Therefore, this confirms the effectiveness of improvements in HOMA-IR and the calibration of the ROC. ROC values ranging from 70 to 80% are considered acceptable, 80 to 90% are considered excellent, and > 90% are considered outstanding [34]. Leptin had the best predictive cutoff value (86% and 92%), whereas Ghrelin had the lowest (70% and 73%). Even the lowest was still considered acceptable. Increasing the power of this study and prolonging follow-up would have possibly changed the sensitivity and specificity to higher cutoff values, which will positively influence the LGr in the future (since they are related to each other). Changing the false-positive and -negative results on the lowest predictive values of ghrelin, GLP-1, PYY, and RBP4 is important because these are on the lower end of acceptability. However, they are still contributing significantly to the care process.

### RBP4

There is increasing evidence that RBP4 induces IR and is closely related to T2DM, obesity, and MBS [35, 36]. In this study, preoperative RBP4 levels were significantly higher in group B participants compared with those of group A (*p* = 0.005). This rise could account for the development of IR by the downregulation of GLUT4 in adipocytes [37, 38]. Results of the present study agreed with those of other studies reporting that decreased RBP4 is associated with changes in BMI after bariatric surgery [39]. Jüllig et al. [40] reported that the decrease in RBP4 levels after bariatric surgery could be due to decreased nutrient absorption resulting in reduced foregut absorption of retinol and the expression of RBRP2 (RBP4 receptors are expressed primarily in the liver, small intestine, and adipocytes). However, multiple logistic regression analysis did not reveal a possible role of RBP4 in predicting the effect on HOMA-IR after bariatric surgery. In addition, the ROC analysis showed a 75% sensitivity and 70% specificity, which was significantly higher than that of insulin, leptin, and LGr. In contrast, Haider et al. found that a decrease in RBP4 might contribute to improvement in IR [39].

The first steps towards new predictive values are shown in this study and are promising for the future.

### Limitations of the Study

This study tested predictive values only in patients who underwent an LSG procedure. The effect of bariatric metabolic surgery using other procedures on the same predictive values should be investigated, for better decision-making. Furthermore, a small sample size could affect the predictive ability of the results since more inclusions give the possibility to include more variables which could help develop new models. Nevertheless, this study was powered well for the expected difference in HOMA-IR. Therefore, it provides a framework for further investigations of the predictive value of these biomarkers to evaluate patients with obesity and IR or T2DM obese or overweight patients who would therapeutically benefit from surgery and treatment decision.

## Conclusion

Preoperative BMI, leptin, ghrelin, LGr, and increased triglycerides have a predictive value on higher postoperative, non-improved patients with HOMA-IR (≥ 2.5). Therefore, an assessment of metabolic biomarkers can help decision on treatment/extra therapy and outcome before surgery in patients who may have insufficient weight loss. All ROC sensitivity and specificity cutoff values of the biomarkers confirmed the postoperative biomarkers in the HOMA-IR < 2.5 group.

## Appendix 1 International Diabetes Federation (IDF) Criteria

Presence of three or more, of the following components was necessary for diagnosis: (1) waist circumference ≥ 94 cm in males and ≥ 80 cm in females, (2) serum triglyceride level ≥ 150 mg/dL, (3) low serum HDL cholesterol concentration < 40 mg/dL in males and < 50 mg/dL in females, (4) systolic blood pressure ≥ 130 mm Hg or/and diastolic blood pressure ≥ 85 mm Hg or previously diagnosed hypertension, (5) fasting plasma glucose ≥ 100 mg/dL.

## Appendix 2 Hormonal Measurements

Serum glucose, total blood cholesterol, high-density lipoprotein (HDL) cholesterol, and triglycerides were measured enzymatically on a Hitachi 7180 Biochemistry Automatic Analyzer (Hitachi, Japan). While low-density lipoprotein (LDL) cholesterol was afterwards calculated using Friedewald’s formula. Fasting insulin levels were measured using ELISA (EIA-2935) (DRG International, Inc. Springfield NJ, USA).

Serum ghrelin was measured using ELISA Kit (Cloud-Clone Corp; cat no: E-01720hu) (W. Fernhurst Dr., Unit 2201, Katy, TX 77,494, USA)). Serum leptin was evaluated by ELISA Kit (Cloud-Clone Corp; cat no: E-00916hu) (TX 77,494, USA). Leptin/ghrelin ratio was calculated in arbitrary units as leptin in nanogram per milliliter multiplied by 10^3^ and divided by ghrelin in picograms per milliliter.

Glucagon peptide 1 quantified using ELISA Kit (Cloud- Clone Corp; Cat no: E-00658hu) (TX 77,494, USA). Human Peptide YY measured by ELISA Kit (Cloud-Clone Corp; Cat no: E-01191hu) (TX 77,494, USA). Serum retinol-binding protein 4 determined by ELISA kit (Cloud-Clone Corp; cat no: SEA929Hu) (TX 77,494, USA).

Homeostasis model assessment of insulin resistance (HOMA-IR) was used to evaluate insulin resistance (fasting serum insulin (μIU/mL) × fasting plasma glucose (mmol/L)/22.5).
